# Suggestions to the Lords

**Published:** 1891-01-17

**Authors:** 


### The Hospital

A Weekly Journal of

Science, Medicine, Nursing, and Philanthropy.

*For Hospitals, Asylums, Infirmaries, Dispensaries, Medical Practitioners, Students, Nurses, and the Charitable Public.*

Vol. IX.—No. 225.] [January 17, 1891.

### SUGGESTIONS TO THE LORDS.

If a Royal Commission, or a Popular Commission, were appointed to sit upon the Constitution of this country, there would be no little fluttering of hearts in certain quarters. What everybody would realise, and most of all the House of Peers, would be this, that something significant was about to happen, and that every man with a stake in the country had better begin to use his brains. Now, small things may be compared with great; but though Britain with her Constitution is great, Hospitals and their affairs are not small. The Committee appointed by the House of Lords does not seem to be aware that the work it has undertaken is of great moment. If Lord Sandhurst had been in her Majesty's Army, and had been asked to take five thousand men and bring certain portions of savage Africa into subjection, he would have given his mental organisation a shake, opened his eyes, pulled himself together, made a plan of campaign, and carried it out with resolution, promptitude, and success.

The application of all this is plain. The hospitals of this country—and we will ask Lord Sandhurst and his Committee to take a note of this statement—are of infinitely greater importance to all classes of people in this country than is the subduing of any part of Africa not already under British rule. For the most part they are voluntary institutions; they have the right of self-government; their constituents, if they so choose, can afford to defy the interference of any external body, however constituted; those constituents, if put upon their high British mettle, will defy the interference of any and every external body if it be meddlesome, irritating, inconsequent, and tending to no useful practical result. English men and women are extremely patient and forbearing. They respect men of position, and venerate authority. But they are certainly not fools.

Floundering about all over the place, like a seal on an iceberg, is not the kind of proceeding which commends itself to the vigorous practical judgment of the average Englishman. Your Englishman dearly loves a horse. It is worth while to enquire for a moment why he loves a horse. The animal has form, and pace; he can go; he can fence; he can be first in at the death. The form is something, but the pace is everything. Lord Sandhurst and his fellow committee-men understand these things well. We have seen their form already as a Hospitals Commission; and it is not good. We have seen their pace, too, and it is simply indescribable in words. In certain parts of England, thirty years ago, there were coal pits which yielded an exceedingly dull and slow burning kind of coal. Each pit was worked by means of a windlass, and at the end of the projecting arm there was generally a miserable old screw of a horse which would have been well sold if the owner of the pit could have got thirty shillings for him. The business of the animal was to go round and round and round in an eternal circle every day, and all the days of his life; and the result of his labours was to bring up, every quarter of an hour, about a bushel of a kind of coal so bad that people gradually ceased to buy it, and the pits were ignominiously closed. Such a gin-horse as this the Lords' Committee threatens to be; such poor results it seems destined to bring forth; and to such an ignominious end it will certainly come unless a new departure be speedily made at its resumed sittings.

What, after all, is it that the public want the Lords' Committee to do? Reasonable men wish for two things: first, that the Committee shall find out whether or not the hospitals are worked as well and as efficiently as they ought to be; and, secondly, that if they are not so worked, their faults shall be pointed out, and authoritative suggestions shall be made for their reform. An enquiry of this kind arranges itself easily under about half a dozen heads. First, and chiefly the financial question demands investigation; for money, in civilised countries is the very life-blood of all public movements. How do the hospitals get their money: and how do they spend it? This is the double-barrelled enquiry which covers almost the whole ground; and to this nine-tenths of the energy of the Committee should be devoted.

Next in importance to the question of finance is that of the patients and their treatment. Who are the patients? Why do they need hospital treatment? On what principal are they selected? What measure of medical and surgical skill is at their service? How are they nursed? How are they housed? How are they fed? These are fundamental enquiries: and the answers to them can be obtained as easily and certainly as the return of a cricket ball from a skilful batsman.

After the patients come the officers, honorary and paid. Hundreds, perhaps thousands, of more or less eminent medical men give their services to hospitals without fee or reward. This is a circumstance almost without parallel in any other profession. But because it is so extraordinary it should be the more keenly inquired into. Are the best interests of the patients fully conserved by such an arrangement? Does it utilise the resources of the hospital in the most efficient way? Are there any persons outside the hospital who are injured by this system? And if there are, can their grievances be discovered and removed? These are questions of deep significance and wide extent: and they affect large numbers of people who help to constitute the backbone of the culture and character of this country. After the honorary staff come the paid medical officials. These are relatively less important. But following upon them again are the Secretaries and clerks. The Secretaries as the official heads of hospitals, are, in actual practice, the most important of all the persons connected with them. The "getting" and the "spending" in the last analysis, appertain to them. Into the appointment, and pay, and powers of these officials full investigation should be made. Next after these again are the matrons, sisters, and nurses. The matrons occupy peculiar positions; they have large responsibilities; and they have and must have, corresponding powers. Responsibility without power is both an absurdity and an iniquity. Is there any man in the world who will order a servant to walk five miles an hour, and first cut off his legs before the journey is begun? Matrons' work, without powers, is impossible to be done. If the matrons have proved themselves unequal to the demand made upon them, then other arrangements must be made. But as long as the responsibility is placed upon their shoulders, it is ridiculous to attempt to deprive them of adequate powers. The sisters and nurses play a part in the life of the hospital which is hardly second in importance to that of the physicians and surgeons themselves. That their condition should be fully investigated, and that every provision should be made which will ensure their happiness and well-being, and also their efficiency, is a proposition which hardly needs to be affirmed. By all means get at the facts. But we submit that if we wish to find out whether or not Lord Sandhurst's domestic establishment is well conducted, we shall not best attain our end by hunting up every discharged, unsuitable, or inefficient servant who may have been in his employ during the last ten years.

Finally, hospitals are managed by a system of committees, and the committees are chosen from among the life governors and others who furnish the funds of the institutions. In other words, voluntary hospitals are administered by those who spend the monies which they themselves have provided. What better guarantee than this could we have that the money will be well spent? What system of management would any sane person be likely to propose which would work better than that which secures the spending of money by those who have contributed it out of their own pockets? The truth of the matter is, that if the Lords could elaborate from existing methods one system of management, which could be more or less uniformly applied to all hospitals; and, more especially, if they could agree to recommend a single and uniform system of keeping accounts, they would do a really valuable work, and practically secure all the reforms that are needed. But if they sit to hear all the complaints which all the discontented and discharged hospital officials of the last ten or fifteen years are disposed to pour into their ears, they will be like a hen painfully incubating a nestful of stone eggs: they will sit for ever without hatching any chickens. The truth is the Lords' Committee is now on its trial, and the course taken by it on the renewal of the enquiry will do much to make or mar, not only its own reputation, but the reputation of each of its members from the Chairman downwards.

